# What are filling (volumizing) threads?

**DOI:** 10.1111/srt.13658

**Published:** 2024-03-21

**Authors:** Kyu‐Ho Yi

**Affiliations:** ^1^ Division in Anatomy and Developmental Biology Department of Oral Biology Human Identification Research Institute BK21 FOUR Project Yonsei University College of Dentistry Seoul South Korea; ^2^ Maylin Clinic (Apgujeong) Seoul South Korea

**Keywords:** facial aging, facial contour enhancement, lifting procedures, thread characteristics, volumizing threads

## Abstract

Facial aging prompts a shift in the demands for lifting procedures, transitioning from targeted improvements in younger individuals to overall facial contour enhancements as skin elasticity declines in later years. This paper examines the evolution of PDO volumizing threads, delineating their development from initial limitations to contemporary innovations aimed at addressing tissue deformation and maintaining thread integrity post‐insertion. Categorizing these threads based on elasticity, shape, and functionality underscores their versatility and application nuances, catering to specific wrinkle correction, contour sculpting, and facial volume restoration. The discussion emphasizes the pivotal role of thread characteristics in achieving optimal outcomes while minimizing potential complications. By delving into historical contexts, mechanisms, effectiveness, and thread classification, this paper equips practitioners with a comprehensive understanding to make informed decisions in selecting threads for volumizing thread procedures. Recommendations for future research directions, including long‐term safety assessments and patient‐specific outcomes, seek to enhance the clinical utility and applicability of this analysis.

## 1 INTRODUCTION

Examining the age‐related demands of patients seeking lifting procedures, it becomes apparent that younger individuals typically desire improvements in facial contouring or skin sagging within specific facial areas rather than overall wrinkles, as their overall skin laxity or wrinkles tend to be minimal.^1^, ^2^, ^3^ As individuals approach their forties to fifties, there is a noticeable decline in skin elasticity, leading to the onset of wrinkles, prompting the need for enhancements in the overall facial contour rather than specific area improvements based on inherent skeletal structure and the condition of skin and connective tissues. This phase is marked by a reduction in the deep fat layers responsible for maintaining facial volume, coupled with the laxity of ligamentous tissues that prevent skin and tissues from sagging, resulting in a gradual widening or expansive appearance of the face. Additionally, collagen and elastin fibers within the skin decrease, sebum secretion diminishes, and the skin thins out, exacerbating skin sagging and accelerating changes in facial structure. Therefore, the physical effects of lifting procedures become essential for improving such facial contours. Post‐melting thread lifting procedures, the histological effects of the melting threads enhance skin elasticity and tone, improve wrinkles, and concurrently ameliorate blood circulation in the inserted areas, potentially yielding a whitening effect.^4^, ^5^, ^6^

Efforts to provide volumizing effects using threads have persisted since the era when only fine simple PDO monofilaments (mono‐threads) were available.^7^, ^8^, ^9^ However, even with multiple fine threads inserted, achieving adequate volume was challenging. Although the formation of capsules around the threads post‐insertion might contribute to additional volume, this effect is generally insignificant. Moreover, the drawback of inserting multiple fine threads involves repeated needle insertions, often resulting in bruising and swelling. Threads designed to address these shortcomings are those aimed at providing volumizing effects.

The initial versions of volumizing threads involved threads curled to form a swirl‐like shape around the needle. However, once these threads entered the tissue, they eventually assumed a shape similar to simple PDO threads due to the surrounding pressure, resulting in limited efficacy. Subsequently, attempts were made to twist threads into a cylindrical shape to maintain the thread's form within tissues post‐insertion. However, these threads also faced issues with decreased elasticity, leading to compression by surrounding tissues post‐insertion. To address this, volumizing threads were developed, gathering multiple threads within a single cannula for simultaneous insertion.

Another type of volumizing thread aimed to maintain the thread's original form without being compressed by external pressure, not by increasing the number of thread strands but by enhancing the thread's intrinsic elasticity. Initially, volumizing threads were crafted in a mesh form, adjusting the elasticity of the threads in cylindrical shapes to prevent deformation post‐insertion into the skin. This design facilitated the easy adherence of collagen‐regenerating fibrous tissues within the internal cylindrical space.

Subsequent to the shortcomings of swirl‐shaped threads being easily compressed within the facial tissue, newer threads were developed that increased the intrinsic elasticity of coiling threads, making them rigid and unyielding upon insertion. These threads could maintain their coil form post‐insertion, resembling a moldable substance, contributing to facial reshaping within the tissue structure.

**FIGURE 1:**
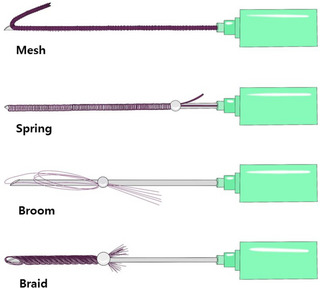
The figure illustrates four distinct types of PDO volumizing multi‐threads designed to achieve a filling effect: the mesh type, spring type, broom type, and braided screw or twisted coil type.

Threads manipulated to possess heightened elasticity not only provide volume but also allow shaping of tissue within the skin, resembling a formative substance. However, excessively resilient threads may lead to skin irregularities, especially if the skin is thin or if the threads are positioned closer to the skin's surface, potentially causing a palpable sensation and surface irregularities. Additionally, if excessively firm volumizing threads are inadvertently placed near sensory nerves, they may induce discomfort. Hence, it is challenging to unequivocally advocate for a particular type or intensity of volumizing thread. Instead, understanding the types and characteristics of each volumizing thread based on the manufacturing approach is crucial for selecting an appropriate thread with the right shape and elasticity, aligned with the objectives of the procedure (Figure 1).

### 1.1 Mechanism and effectiveness

The procedural effects of PDO Volumizing Multi‐Threads can be broadly categorized into two mechanisms based on their actions following insertion. Firstly, volumizing threads designed by combining multiple fine monofilaments aim to maximize the effects of conventional PDO monofilaments.^10^, ^11^ These threads do not significantly increase the overall elasticity of the threads but instead provide a relatively soft cushion‐like role. They are typically utilized to fill deep‐seated visible wrinkles or smooth out skin surfaces that appear irregular due to wrinkles. Their lower elasticity allows for proximity to the skin without causing significant bulging or palpable sensations, owing to their composition of multiple fine monofilaments.^12^, ^13^, ^14^ However, unless numerous monofilaments are inserted, these threads, by their stacking nature, cannot generate a substantial overall volume or distinctive shapes, making it challenging to provide substantial volume to the skin or create precise shapes using threads alone. Nonetheless, these volumizing threads serve as a valuable option when correcting deeply etched wrinkles that are challenging to address solely with fillers or smoothing out skin unevenness caused by expression lines, thereby adding suitable volume to impart a firm sensation to the skin.

**FIGURE 2:**
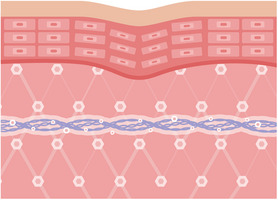
The figure demonstrates the physical support provided by PDO volumizing threads when utilized in combination treatments with HA fillers. This support offers a scaffold effect, delivering mechanical reinforcement that facilitates attachment structures for regenerative cells and various biochemical factors. Moreover, these threads offer physical support, particularly in soft areas like the nose, and can be applied in mesh, plate, or other forms. Acting as collaborators for biological response, these threads function as structural backbones, concentrating active materials within target areas. Furthermore, they induce a foreign body reaction, which aids in attracting biochemical factors. Lastly, these threads serve as stimulants by inciting an inflammatory response, consequently amplifying responses and synergizing with attracted biochemical factors.

For sculpting specific areas or achieving distinct contours using volumizing threads, it is preferable to use threads with good elasticity. Typically, these threads feel firm upon touch and resist compression, making them more suitable for insertion deeper into the subdermal layer or areas beyond. When addressing wrinkles, these threads are beneficial for revitalizing more extensive and deeply furrowed areas, such as rejuvenating the nasolabial fold adjacent to the nasal tip or the prejowl sulcus inside the corner of the mouth, as opposed to fine, linear wrinkles. Another benefit of using firm volumizing threads is their capacity to restore facial contours across various facial areas, acting as a substitute for fillers to enhance facial features. When used in conjunction with HA fillers, these threads can maximize the procedural effects depending on the context (Figure 2).^8^, ^9^, ^15^

### 1.2 Classification volumizing threads

**FIGURE 3:**
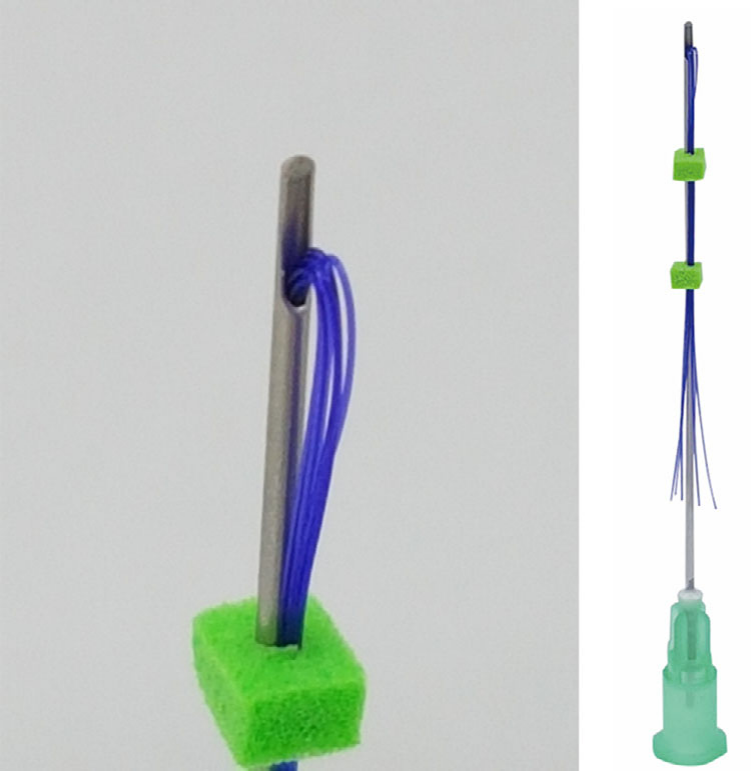
The figure displays the broom type of PDO volumizing threads.

**FIGURE 4:**
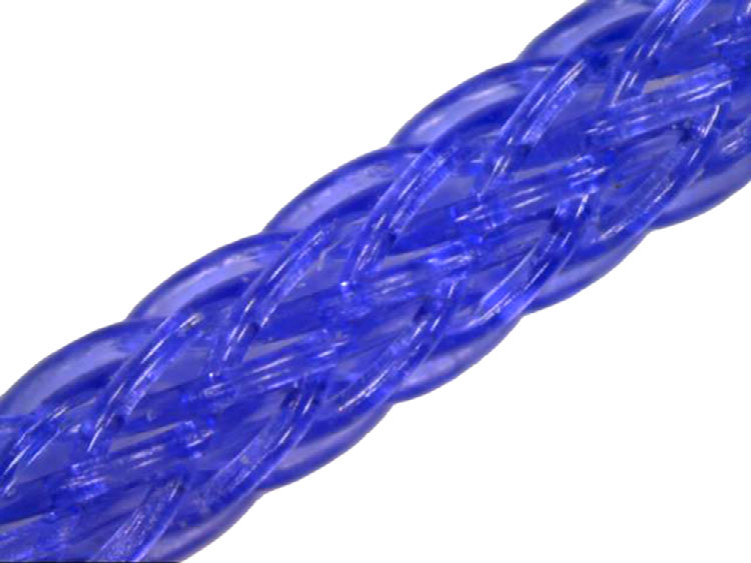
The figure exhibits the braided screw or twisted coil type of volumizing PDO threads.

The PDO Volumizing Multi‐Threads can be broadly categorized into two types based on their established configurations. One category comprises threads arranged in a broom or rope‐like manner, featuring stacked layers of fine threads. These volumizing threads, such as the broom type or braided screw or twisted coil type, typically consist of multiple layers of fine PDO threads gathered or twisted to form a broom‐like structure (Figure 3) or further coiled into a screw‐like or coil‐shaped pattern (Figure 4). Threads of this kind, due to their layered composition, can provide a degree of volume upon insertion. Each thread is slender, preventing visibility of the thread on the skin surface or the outline of threads beneath the skin, thus avoiding any palpable contours. However, as these threads merely conglomerate several fine threads without forming a distinct shape, they can fill voids underneath the skin but possess limitations in sculpting defined contours. Consequently, these volumizing threads prove advantageous for addressing deeply etched wrinkles on the skin surface that are challenging to fill with fillers alone. Their utility extends to various areas such as forehead furrows in men, glabellar lines, periorbital and infraorbital areas, nasolabial folds, upper and lower lips, static lines around the mouth, and horizontal neck lines.^16^, ^17^, ^18^, ^19^, ^20^

**FIGURE 5:**
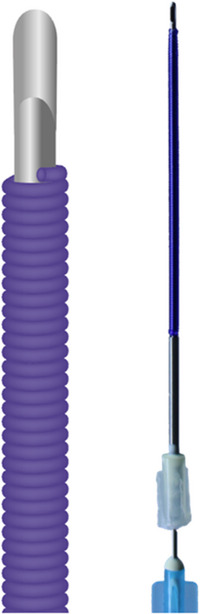
The figure demonstrates the spring type (Countourel (Licellvi), J World Co., Ltd., Korea). This volumizing spring thread, composed of a coil shape, retains a cylindrical or tube‐shaped form inside, allowing for collagen production within it, thereby enhancing the effectiveness of volumizing.

The other type encompasses volumizing threads with consistent shapes resembling a cannula or needle, typically elongated and rounded cylindrical forms. Among these, the most commonly used threads include the spring type and cylindrical mesh type. Contemporary spring‐type threads typically exhibit a dual‐needle structure, with PDO threads enclosed within a larger needle. Inserting the larger needle into the treatment area and subsequently retracting it leaves behind the spring‐shaped thread within the smaller needle, which, when carefully removed, leaves only the volumizing thread beneath the skin. In contrast to conventional spring‐type threads, these modern threads possess a robust spring shape that remains intact post‐procedure without compressing the thread's form (Figure 5).

**FIGURE 6:**
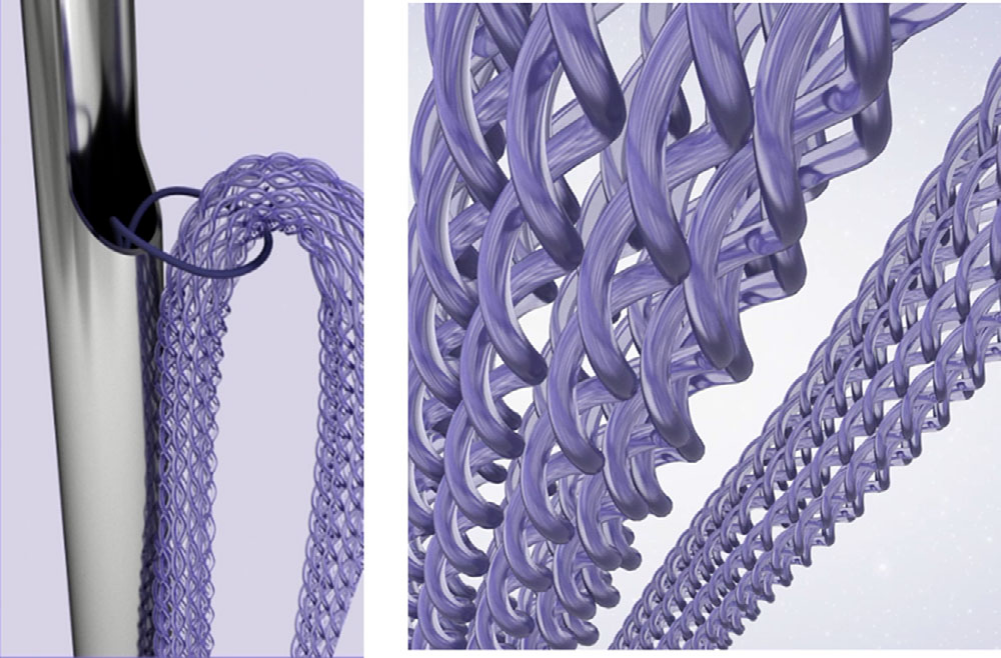
The figure illustrates the cylindrical mesh type of PDO volumizing thread.

The cylindrical mesh type, on the other hand, involves threads twisted together to form a cylindrical shape, creating an internal hollow space allowing tissue growth for additional volumizing effects. Additionally, tissue proliferation occurs around the cylindrical periphery (Figure 6). Typically, spring‐type volumizing threads exhibit a more prominent and rigid structure within the overall volumizing thread compared to cylindrical mesh‐type threads. While this characteristic can maintain the thread's shape well within the skin, it might also present both advantages and disadvantages. If the thread is positioned too close to the skin surface, the relatively rigid spring structure might protrude externally, and if placed near sensory nerves, it might induce temporary discomfort. Therefore, when employing volumizing threads with robust elasticity, considerations regarding the thread's shape, thickness, and firmness are imperative, aligning with the treatment area and objectives, alongside careful selection of insertion depth.

Presently, volumizing threads of similar shapes and configurations can be classified into soft and firmer types by adjusting the thread's intrinsic elasticity. Particularly, threads with heightened elasticity can resist compression beneath the skin, maintaining their shape akin to a moldable substance.

## 2 DISCUSSION

Aging induces a decline in skin elasticity, volumetric fat layers, collagen, and elastin, leading to facial laxity, wrinkling, and changes in facial contours. These aspects underscore the importance of lifting procedures, especially post‐melting thread lifting methods, inducing histological effects that improve skin quality, wrinkles, and circulation in treated areas.

The narrative traces the historical trajectory of efforts to achieve volumizing effects using threads. Early attempts with fine PDO monofilaments faced challenges in providing adequate volume due to structural limitations. Innovations in thread designs aimed to address these issues by focusing on volumizing effects. Various types of volumizing threads are introduced, engineered to resist deformation and maintain their original forms within tissues post‐insertion, reflecting a nuanced understanding of thread characteristics for effective lifting procedures.^6^, ^21^, ^22^, ^23^

Volumizing threads are categorized based on mechanisms and effectiveness, showcasing the diversity in their applications.^24^, ^25^ Threads with multiple fine monofilaments address specific wrinkles and irregular skin surfaces but have limitations in providing substantial overall volume. Threads with better elasticity are highlighted for sculpting specific areas and restoring facial contours, offering versatile applications across different facial regions. Firm volumizing threads, in conjunction with HA fillers, complement each other, enhancing procedural outcomes.

The classification of volumizing threads into distinct categories provides comprehensive insights into their structural compositions and potential utilities. The discussion on broom or rope‐like structures and elongated cylindrical forms offers valuable insights into their respective advantages and limitations, guiding practitioners in making informed decisions during procedures.

The implications of thread characteristics, particularly their elasticity, address the potential drawbacks of overly resilient threads. Emphasis is placed on selecting threads aligned with specific procedural objectives, considering factors such as thread shape, thickness, firmness, and insertion depth to optimize outcomes and minimize potential complications.

Overall, the comprehensive analysis of PDO Volumizing Multi‐Threads spans their historical evolution, mechanisms, effectiveness, and classification, providing practitioners with a robust foundation to make informed decisions in selecting appropriate threads for lifting procedures.

To further enrich understanding, future research directions could encompass long‐term safety assessments, comparative studies of thread types, and advancements in thread technologies. Discussing patient‐specific considerations and outcomes would enhance the paper's applicability in clinical settings.

## CONFLICT OF INTEREST STATEMENT

I acknowledge that I have considered the conflict of interest statement included in the “Author Guidelines.” I hereby certify that, to the best of my knowledge, that no aspect of my current personal or professional situation might reasonably be expected to significantly affect my views on the subject I am presenting.

## Data Availability

Data sharing is not applicable to this article as no new data were created or analyzed in this study.
